# Phytochemical composition and bioactivities of Saudi endemic *Reseda pentagyna* essential oils

**DOI:** 10.1038/s41598-026-42479-y

**Published:** 2026-03-05

**Authors:** Ibrahim M. Aziz, Rawan M. Alshalan, Amal Khalaf Alghamdi, Mohamed A. Farrag, Sahar Abdulaziz AlSedairy, Abdulaziz M. Almuqrin

**Affiliations:** 1 Department of Botany and Microbiology, College of Science, King Saud University, P.O. Box 2455, Riyadh, 11451 Saudi Arabia; 2 Department of Food Sciences and Nutrition, College of Food and Agricultural Sciences, King Saud University, P.O. Box 2460, Riyadh, 11451 Saudi Arabia; 3 Department of Clinical Laboratory Sciences, College of Applied Medical Sciences, King Saud University, P.O. Box 10219, Riyadh, 12372 Saudi Arabia

**Keywords:** *Reseda pentagyna*, Essential oils, Alternative therapy, Antioxidant, Antibacterial resistance, Antidiabetic, Saudi medicinal plants, Biochemistry, Biotechnology, Cancer, Drug discovery, Microbiology, Plant sciences

## Abstract

*Reseda pentagyna*, a medicinal plant native to Saudi Arabia, has little scientific evidence of its phytochemical composition and biological capabilities. Despite its potential therapeutic benefits, the *Resedaceae* family has received little research attention. The purpose of this study was to analyze the phytochemical composition of *R. pentagyna* leaves essential oil (RPLEO) from Abha, Saudi Arabia, and evaluate its antioxidant, antibacterial, antidiabetic, and cytotoxic effects. The essential oils (EOs) were extracted via hydro-distillation and yielded 4.75 ± 1.75 (W/V). GC-MS analysis showed a robustly dominated monoterpenoid composition, mainly carvacrol (21.59%) as the predominant compound, followed by thymol (3.62%) and other phenolic constituents. RPLEO exhibits high antioxidant activity (DPPH IC_50_ = 80.13 ± 1.53 µg/mL, ABTS IC_50_ = 92.72 ± 1.34 µg/mL), which explains the high TPC (141.30 mg GAE/g) and TFC (88.34 mg QAE/g). Furthermore, RPLEO showed an antibacterial activity against Gram-negative pathogens (MIC = 6.25–12.50 µg/mL). It also showed notable antidiabetic potential by inhibiting α-amylase (IC_50_ = 82.31 µg/mL) and α-glucosidase (IC_50_ = 87.49 µg/mL) enzymes. Furthermore, it induced considerable cytotoxicity against HepG2 (IC_50_ = 112.34 µg/mL) and MCF-7 (IC_50_ = 126.34 µg/mL) cells, confirmed by the upregulation of pro-apoptotic markers (caspase-3, -8, -9, Bax) and downregulation of anti-apoptotic markers (Bcl-2, Bcl-xL). This study provides the first comprehensive analysis of RPLEO, demonstrating significant bioactivity. Further investigations, including compound isolation, in vivo validation, and clinical translation, are recommended to fully realize its medicinal potential.

## Introduction

Natural products have been the cornerstone of traditional medicine for millennia and continue to play a significant role in modern drug development. ^1^. More than half of the currently licensed medications are derived from or inspired by plant secondary metabolites, which are particularly important in antibacterial, anticancer, and metabolic illness therapy^2^. Despite mounting evidence of its pharmacological potential, the *Resedaceae* family remains one of the most understudied among medicinal plants.

Despite its tiny size, the family *Resedaceae* contains 75–85 species divided into 6–7 genera: *Reseda*,* Ochradenus*,* Caylusea*,* Oligomeris*,* Sesamoides*, and *Randonia*^3^. The species is found predominantly in temperate and subtropical countries, with a strong presence in Mediterranean climates and deserts. Their growth style ranges from herbaceous to shrubby, with opposite leaves and racemose inflorescences containing zygomorphic flowers that typically produce capsular fruits^4^. The majority of *Resedaceae* members have glucosinolates, which have been shown to have chemopreventive activity against several malignancies through the hydrolysis to isothiocyanates^5^. Along with isothiocyanates, *Resedaceae* plants produce typical secondary metabolites such as flavonoids like luteolin and apigenin derivatives with antioxidants and anti-inflammatory properties, phenolic acids with antimicrobial activity against antibiotic-resistant bacteria, and alkaloids with potential neuroprotective activity. Modern scientific research has validated several of these uses and discovered additional therapeutic opportunities, such as diabetes treatment through inhibition of α-glucosidase and antitumor effects by inducing apoptosis in cancer cells. Furthermore, research has shown potential for preventing microbial infections, particularly against Gram-positive bacteria^6^.

*Reseda pentagyna* is a *Resedaceae* herb endemic to Saudi Arabia^7^. It is a native annual herb belonging to the order *Brassicales*, the genus *Reseda*, which contains approximately 65 species worldwide, with a primary distribution in the Mediterranean region and arid regions^8^. *R. pentagyna* is one of just seven *Reseda* species to be reported in Saudi Arabia^7^. This species morphologically has the typical five- to six-toothed capsule and extensive rocky, arid habitat adaptations. Recent genomic studies have revealed *R. pentagyna* to possess a greater genome size compared to that of its relatives, such as *Reseda lutea*, with high heterozygosity, suggesting special evolutionary adaptations to its difficult native habitat^9^. In Saudi Arabia, *R. pentagyna* is primarily located in the mountain ranges such as Abha, Tabuk, and Hijaz^7^. Its restricted distribution in these specific ecological niches indicates both its ecological specialization and priority for conservation, since endemic species of limited range are vulnerable to habitat fragmentation and impacts from climate change. Despite limited studies about *R. pentagyna*, other related *Resedaceae* family species have some bioactive evidence. *Reseda lutea*, for instance, has been found to have potent antidiabetic activity through α-amylase inhibition as well as antioxidant and cytotoxic activities against various cancer cell lines, attributable to its flavonoid content^10^. Consequently, *Ochradenus arabicus*, being another *Resedaceae*, proved to exhibit very high antimicrobial and enzyme inhibitory activity due to its richness in isothiocyanates^6^.

Based on the above studies, it would appear that *R. pentagyna* can be inferred to be supposed to exhibit the same or possibly superior pharmacological characteristics, justifying a wider screening. Existing studies on similar species suggest possible uses in antioxidant, antimicrobial, and antidiabetic treatments, but crucial gaps exist in our knowledge of *R. pentagyna*’s phytochemical content and biological activities. Additionally, the absence of thorough investigations into its mechanistic pathways and sustainable use strategies highlights the importance of targeted research. Closing these gaps would not only enhance our understanding of this species but also benefit the general field of medicinal plant research.

So, the current study intends to offer a complete characterization of *R. pentagyna* leaf essential oils (RPLEO), including detailed phytochemical profiling via gas chromatography-mass spectrometry (GC-MS) and quantification of total phenolic content (TPC) and total flavonoid content (TFC) of phenolic and flavonoid contents. We intend to assess its biological activities, with a particular emphasis on antioxidant capacity, antibacterial activity against clinically relevant infections, and inhibitory effects on carbohydrate-digesting enzymes important for diabetes management. Furthermore, the cytotoxic ability of RPLEO will be tested against chosen cancer cell lines to investigate potential therapeutic uses. Our multimodal strategy aims to overcome existing knowledge gaps while also showcasing *R. pentagyna*’s potential as a source of new bioactive chemicals.

## Results

### Extraction yields

The extraction yield, calculated based on dry matter weight (w/w), demonstrated that the RPLEO extraction process achieved a yield of 4.75 ± 1.75% (v/w).

### Chemical composition of RPLEO

**Fig. 1:**
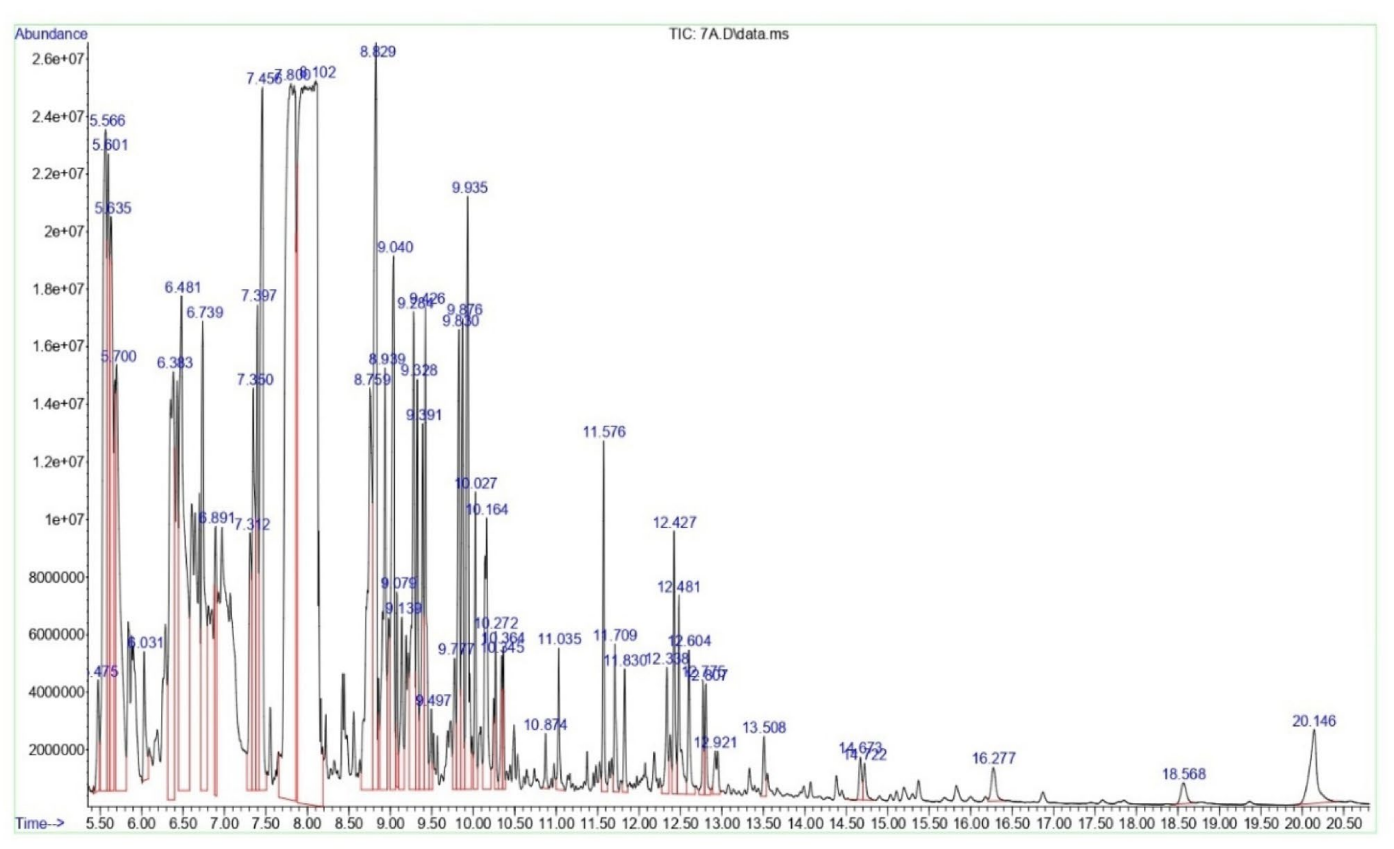
The GC-MS chromatograms of RPLEO. All spectral peaks correlate with the identified chemicals, with a major peak indicating the primary constituent of the extract.

The bioactive components of the RPLEO were identified by comparing the molecular weight, molecular formula, peak retention time, and peak area (%) of the 53 peaks that were obtained with those of the recognized compounds in the NIST database. The profile was characterized by phenolic compounds, with Carvacrol being the most common component, accounting for 21.59%, and thymol (3.62%), which are known for their broad bioactivity. The following components were Benzene, 1-methyl-3-(1-methylethyl) (4.08%), Cyclohexanone, 2-methyl-5-(1-methylethenyl)-, trans-(4.65%), and Phenol, 2-(1,1-dimethylethyl)-5-methyl- (11.48%), as indicated in Table 1; Fig. 1. Compound identification was confirmed by comparing mass spectra to the NIST library (matching factor ≥ 90%) and, where available, to previously published data on *Resedaceae* species [6,9,11]. Compounds without CAS numbers (peaks 32, 41, 43, 46, and 48) were identified using mass spectral fragmentation patterns and compared to literature descriptions of these compounds in other plant species.

**Table 1:** Chemical compounds of RPLEO identified by GC-MS.

Peak	Retention Time (min)	Area	Area%	Name	Molecular formula	Molecular weight	Cas No.	Classification
1	5.477	69,882,449	0.39	α- Terpinolen	C_10_H_16_	136	586-62-9	Monoterpenoids
2	5.566	722,961,088	4.08	m-Cymene	C_10_H_14_	134	535-77-3	Cumenes
3	5.601	427,324,541	2.41	p-Cymene	C_10_H_14_	134	99-87-6	Monoterpenoids
4	5.635	481,488,530	2.71	cis-Sabinene hydroxide	C_10_H_18_O	154	15537-55-0	Monoterpenoids
5	5.7	547,040,864	3.08	Cineole	C_10_H_18_O	154	470-82-6	Oxanes
6	6.031	79,770,540	0.45	cis-β-Terpineol	C_10_H_18_O	154	138-87-4	Monoterpenoids
7	6.383	587,153,503	3.31	Thujone	C_10_H_16_O	152	546-80-5	Monoterpenoids
8	6.481	824,632,667	4.65	trans-Dihydrocarvone	C_10_H_16_O	152	5948-04-09	Monoterpenoids
9	6.739	434,300,513	2.45	(+)-2-Bornanone	C_10_H_16_O	152	464-49-3	Monoterpenoids
10	6.891	134,160,543	0.75	endo-Borneol	C_10_H_18_O	154	507-70-0	Monoterpenoids
11	7.35	303,459,606	1.71	2-isopropyl-4-methylanisole	C_11_H_16_O	164	1076-56-8	Cumenes
12	7.397	292,189,991	1.64	p-tert-Butylanisole	C_11_H_16_O	164	5396-89-4	Phenylpropanes
13	7.456	603,751,221	3.41	methyl-ether Carvacrol	C_11_H_16_O	164	6379-73-3	Monoterpenoids
14	7.8	2,034,819,819	11.48	6-tert-Butyl-m-Cresol	C_11_H_16_O	164	88-60-8	Phenylpropanes
15	8.102	3,825,072,856	21.59	Carvacrol	C_10_H_14_O	150	499-75-2	Monoterpenoids
16	8.759	534,408,874	3.01	Thymol	C_10_H_14_O	150	89-83-8	Monoterpenoids
17	8.829	641,687,739	3.62	Caryophyllene	C_15_H_24_	204	87-44-5	Sesquiterpenoids
18	8.939	365,248,878	2.06	Cis-caryophyllene	C_15_H_24_	204	118-65-0	Sesquiterpenoids
19	9.04	420,987,360	2.37	Aromandendrene	C_15_H_24_	204	489-39-4	Sesquiterpenoids
20	9.079	78,315,491	0.44	Humulene	C_15_H_24_	204	4753-98-6	Sesquiterpenoids
21	9.139	116,582,023	0.65	α-Himachalene	C_15_H_24_	204	3853-83-6	Sesquiterpenoids
22	9.284	396,601,938	2.2	Longifolene-(V4)	C_15_H_24_	204	475-20-7	Monoterpenoids
23	9.328	184,112,872	1.03	β-Himachalene	C_15_H_24_	204	1461-03-6	Sesquiterpenoids
24	9.391	212,011,316	1.19	γ-Cadinene	C_15_H_24_	204	483-74-9	Sesquiterpenoids
25	9.426	276,857,090	1.56	δ-Cadinene	C_15_H_24_	204	483-76-1	Sesquiterpenoids
26	9.497	42,585,778	0.24	α-epi-7-epi-5-Eudesmol	C_15_H_22_O	214	446050-56-2	Sesquiterpenoids
27	9.777	149,466,648	0.83	Isoaromadendrene epoxide	C_15_H_24_O	220.35	104664-42-0	Sesquiterpenoids
28	9.83	283,304,316	1.59	Caryophyllene oxide	C_15_H_24_O	220	1139-30-6	Sesquiterpenoids
29	9.876	241,031,129	1.36	Viridiflorol	C_15_H_26_O	222	552-02-3	Sesquiterpenoids
30	9.936	393,780,315	2.22	1R,7 S, E)-7-Isopropyl-4,10-dimethylenecyclodec-5-enol	C_15_H_24_O	220	81968-62-9	Sesquiterpenoids
31	10.027	134,884,053	0.76	Calarene epoxide	C_15_H_24_O	220	11000-57-0	Monoterpenoids
32	10.164	235,115,344	1.32	9-Isopropyl-1-methyl-2-methylene-5-oxatricyclo[5.4.0.0(3,8)]undecane	C_15_H_24_O	220	NA	Sesquiterpenoids
33	10.345	59,945,098	0.33	γ-costol	C_15_H_24_O	220	6892-80-2	Sesquiterpenoids
34	10.364	56,729,127	0.32	α-Bisabolol	C_15_H_26_O	222	515-69-5	Sesquiterpenoids
35	10.874	24,471,117	0.13	(Z)-α-Atlantone	C_15_H_22_O	218	26611-71-2	Sesquiterpenoids
36	11.035	72,045,180	0.41	trans-α-Atlantone	C_15_H_22_O	218	26611-70-1	Sesquiterpenoids
37	11.576	156,428,873	0.88	2,5,5,8a-Tetramethyl-1,2,3,5,6,7,8,8a-octahydronaphthalen-1-ol	C_14_H_24_O	208	20489-45-6	Alcohols & polyols
38	11.709	83,958,811	0.47	Pimaradiene	C_20_H_32_	272	1686-61-9	Diterpenoids
39	11.83	57,080,890	0.32	Guaia-3,9-diene	C_15_H_24_	204	489-83-8	Sesquiterpenoids
40	12.338	81,099,594	0.45	Guaia-1(10),11-diene	C_15_H_24_	204	107115-83-5	Sesquiterpenoids
41	12.427	132,131,380	0.74	Geranyl-α-terpinene	C_20_H_32_	272	NA	Sesquiterpenoids
42	12.481	136,408,935	0.77	trans-Geranylgeraniol	C_20_H_34_O	290	24034-73-9	Diterpenoids
43	12.604	88,826,732	0.51	Methyl octadeca-9-yn-11-trans-enoate	C_19_H_32_O_2_	292	NA	Fatty acid esters
44	12.775	56,775,777	0.32	Retinoic acid	C_20_H_28_O_2_	300	302-79-4	Retinoids
45	12.807	57,426,929	0.32	Adrenosterone	C_19_H_24_O_3_	300	382-45-6	Androstane steroids
46	12.921	50,437,576	0.28	2-[4-methyl-6-(2,6,6-trimethylcyclohex-1-enyl)hexa-1,3,5-trienyl]cyclohex-1-en-1-carboxaldehyde	C_23_H_32_O	324	NA	Retinoids
47	13.508	37,963,904	0.21	Pregnan-20-one, 21-(acetyloxy)-3-hydroxy-, (5α)-	C_23_H_36_O_4_	376	587-64-4	Pregnane steroids
48	14.673	32,537,779	0.18	Oct-5-en-2-ol, 8-(1,4,4a,5,6,7,8,8a-octahydro-2, 5, 5, 8a-tetramethylnaphth-1-yl)-6-methyl-	C_23_H_40_O	332	NA	Diterpenoids
49	14.722	30,577,496	0.17	Erucylamide	C_22_H_43_NO	337	112-84-5	Fatty amides
50	16.277	44,430,559	0.25	Lupeol	C_30_H_50_O	426	545-47-1	Triterpenoids
51	18.568	33,262,042	0.18	Spirost-8-en-11-one, 3-hydroxy-, (3β,5α,14β,20β,22β,25R)-	C_27_H_40_O_4_	428	1260-07-1	Oxosteroids

### TPC and TFC of RPLEO

**Fig. 2:**
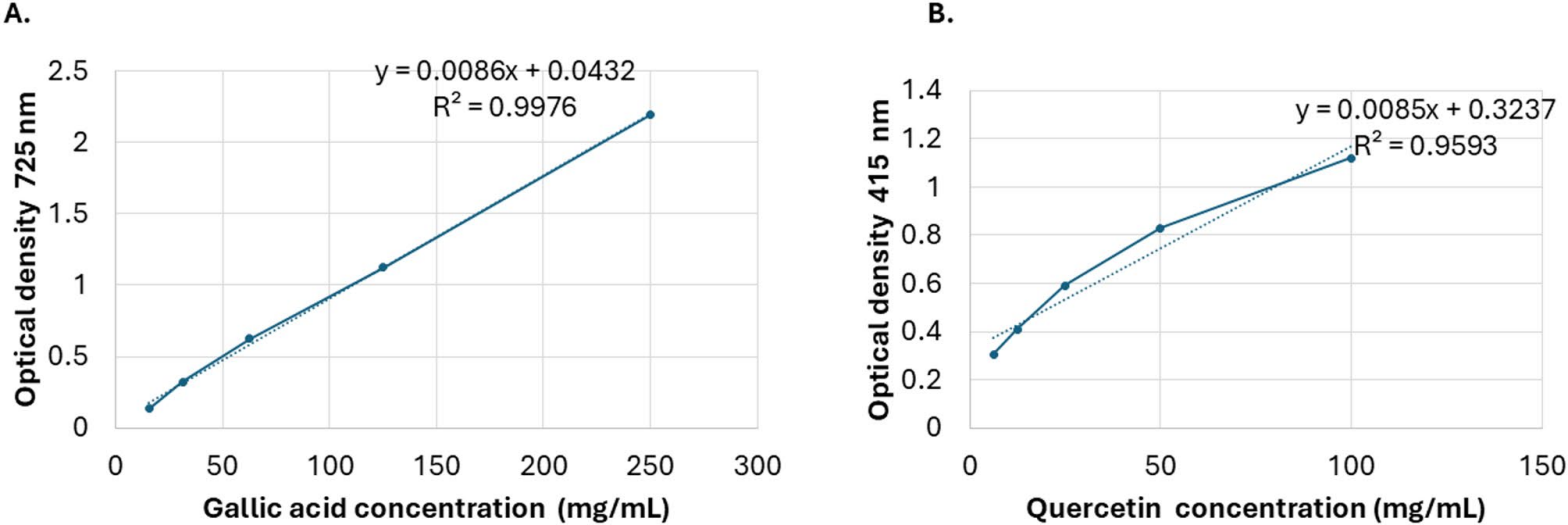
(**A**), Standard curve of gallic acid for TPC estimation(**B**), Standard curve of quercetin (TFC).

By applying the Folin-Ciocalteu method and gallic acid as a reference, the TPC of methanolic extracts was calculated. The RPLEO revealed a TPC of (141.30 ± 1.72 mg Gallic acid equivalent (GAE)/g dry weight of the extract). The calibration curve for gallic acid showed excellent linearity with an R^2^ = 0.9976 (Fig. 2A). That indicates a robust synchronization between the findings of both techniques and highlights their reliability. Using quercetin as a reference, the TFC of RPLEO was determined using the Aluminum chloride (AlCl_3_) colorimetric technique; the total flavonoid content was found to be 88.34 ± 1.34 mg quercetin equivalent (QAE)/g dry weight of extract. The quercetin standard curve showed an R^2^ of 0.9593, which demonstrates acceptable linearity and accuracy for TFC, Fig. 2B.

### Antioxidant activity

**Fig. 3:**
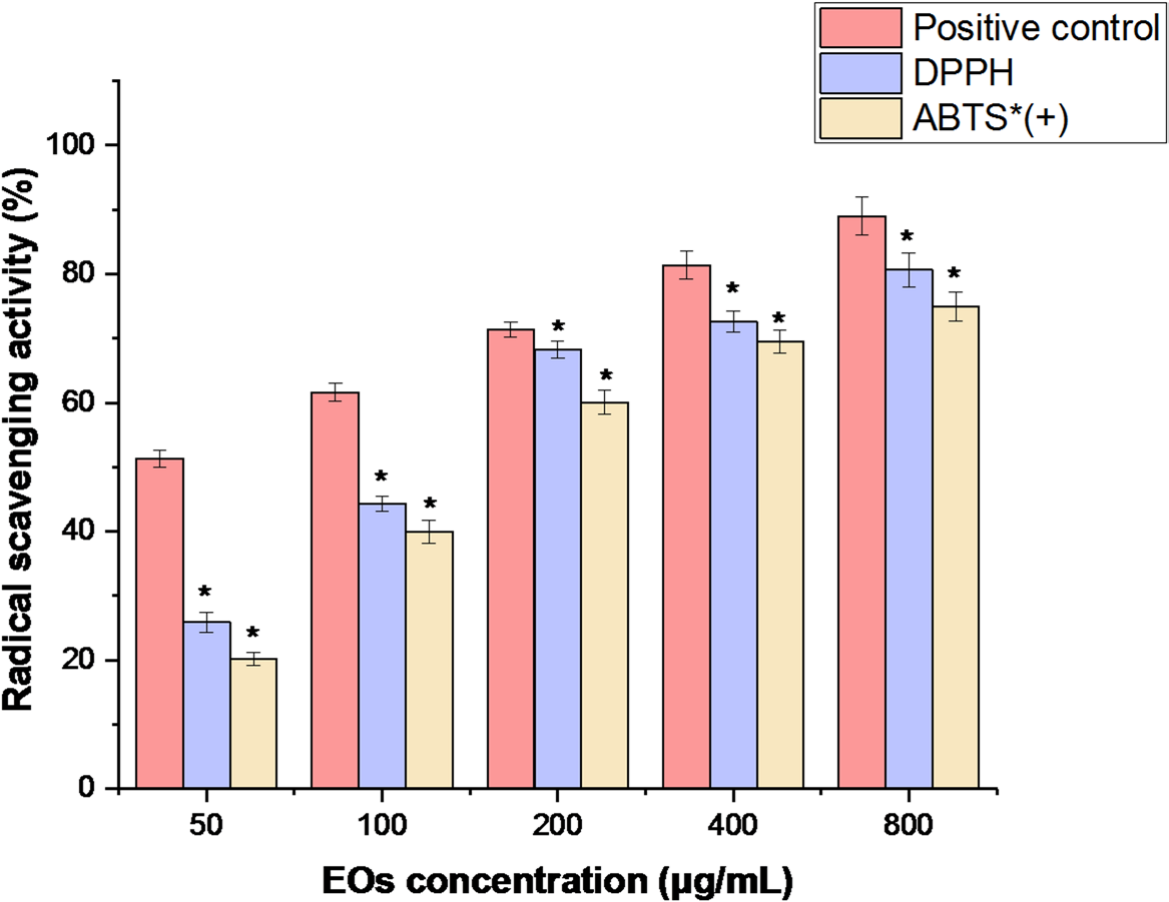
Antioxidant activity (DPPH and ABTS scavenging activity) of methanol extract from the flowers of *M. recutita* L. at various concentrations (50–800 µg/mL). The results are the mean values of three replicates.

The antioxidant activity of EO was assessed using two techniques: 1,1-diphenyl-2-picryl hydrazyl (DPPH) *assay* and 2,2-azino-bis (3-ethylbenzothiazoline-6-sulfonic acid) (ABTS) assay. As illustrated in Fig. 3, at a low concentration of 50 µg/mL, the RPLEO, using ascorbic acid as a positive control, exhibited strong radical scavenging activity in both the DPPH and ABTS assays. Higher antioxidant activity was demonstrated at high doses of RPLEO in both the DPPH and ABTS findings, with IC_50_ values of 80.13 ± 1.53 µg/mL and 92.97 ± 1.34 µg/mL, respectively. In contrast to the positive control (IC_50_ = 30.61 ± 2.31 µg/mL), EO was significantly (*p* < 0.05) less effective.

### Antibacterial effects of RPLEO

The disc diffusion technique, minimum inhibitory concentration (MIC) and bactericidal concentration (MBC) were used to evaluate RPLEO’s antibacterial activity compared to the positive control (25 µg/mL of chloramphenicol). The results demonstrated that RPLEO inhibited most of the Gram-positive and Gram-negative bacterial growth in a dose-dependent manner. Gram-negative bacteria were more susceptible to RPLEO, with MIC values ranging from 6.25 ± to 12.50 µg/mL, while Gram-positive bacteria showed MIC values of 12.50 to 50.00 µg/mL (Table 2).

**Table 2:** Antibacterial activities of RPLEO as evidenced from the zones of inhibition (ZoI) (mm), MIC (µg/mL**)**, and MBC (µg/mL).

Bacterium/ Dilution	Positive control	800 µg/mL	400 µg/mL	200 µg/mL	100 µg/mL	MIC (µg/mL)	MBC (µg/mL)
*Staphylococcus aureus*	26 ± 0.00	19 ± 0.00 *	14 ± 0.00 *	11 ± 0.00 *	9 ± 0.00 *	25.00 ± 0.00	50.00 ± 0.00
*Enterococcus. faecalis*	27 ± 0.00	18 ± 0.00 *	13 ± 0.00 *	10 ± 0.00 *	8 ± 0.00 *	12.50 ± 0.00	25.00 ± 0.00
*Bacillus subtilis*	24 ± 0.00	13 ± 0.00 *	9 ± 0.00 *	7 ± 0.00 *	0 ± 0.00 *	50.00 ± 0.00	100.00 ± 0.00
*Escherichia coli*	23 ± 0.00	19 ± 0.00 *	17 ± 0.00 *	15 ± 0.00 *	9 ± 0.00 *	6.25 ± 0.00	12.50 ± 0.00
*Klebsiella pneumoniae*	22 ± 0.00	18 ± 0.00 *	16 ± 0.00 *	15 ± 0.00 *	11 ± 0.00 *	12.50 ± 0.00	25.00 ± 0.00
*Pseudomonas aeruginosa*	25 ± 0.00	19 ± 0.00 *	18 ± 0.00 *	15 ± 0.00 *	13 ± 0.00 *	12.50 ± 0.00	25.00 ± 0.00

The reported values are shown in triplicate as mean ± SD. Statistical analysis was performed using one-way analysis of variance (ANOVA) followed by Dunnett’s post-hoc test. The results demonstrate a statistically significant decrease from the positive control (25 µg/ml of chloramphenicol), indicated by (* = p < 0.05).

### In vitro α-amylase and α-glucosidase inhibition activities

**Fig. 4:**
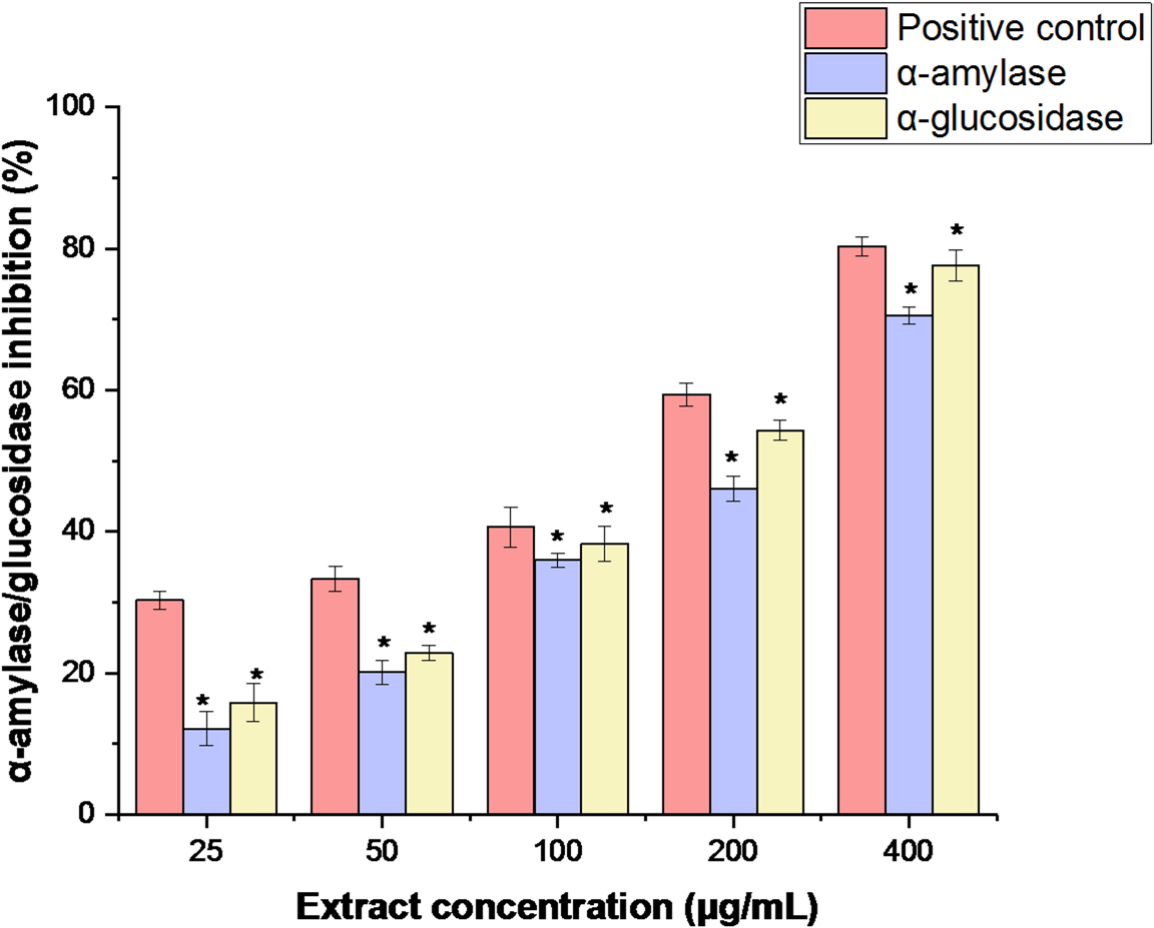
*α*-Amylase and *α*-glucosidase inhibitory activities of methanol extract from the RPLEO at various concentrations (25–400 µg/mL). The results are the mean values of three replicates.

The in vitro inhibition of the α-amylase and α-glucosidase enzymes was used to measure the antidiabetic effect of RPLEO. The findings showed that RPLEO significantly reduced the activity of α-amylase and α-glucosidase at 100 µg/mL and above (*p* < 0.05), but less than the positive control (acarbose); RPLEO concentrations further exerted a gradient inhibition of α-amylase and α-glucosidase enzymes with increasing concentration (Fig. 4). Additionally, with IC_50_ values of 87.49 ± 1.19 and 82.31 ± 1.28 µg/mL for α-glucosidase and amylase, respectively, RPLEO demonstrated greater potential antidiabetic efficacy.

### Cell cytotoxicity and apoptosis markers

**Fig. 5:**
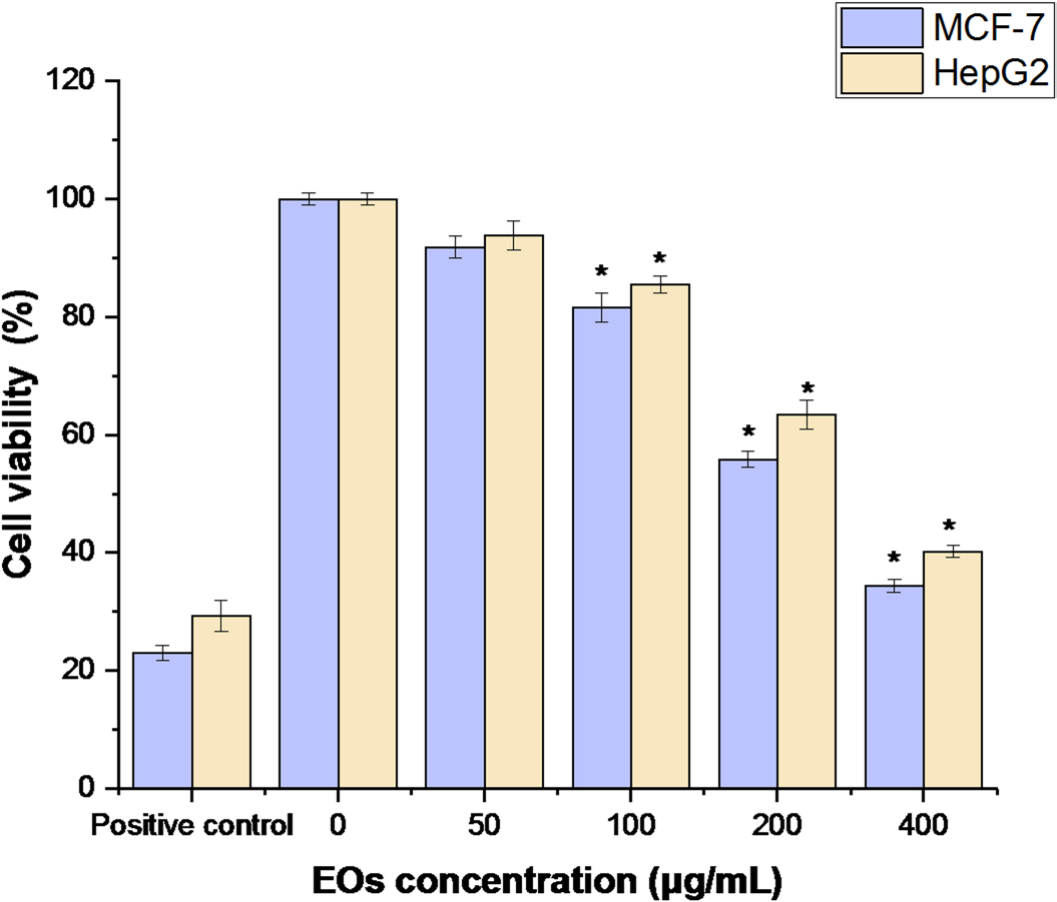
Effect of EO on cell viability. The cells were exposed to the specified concentration (0–400 µg/mL) of RPLEO for 24 h, and the viability of the cells was determined by the MTT assay. Cell viabilities are shown as percentages, and the untreated cells were regarded as 100% viable. Mean ± standard deviation (SD) is presented from three independent experiments, (* = *P* < 0.05 compared to non-treated cells (negative control)).

The A 3-(4,5-dimethylthiazol-2-yl)-2,5-diphenyl-2 H-tetrazolium bromide (MTT) assay was used to quantify the cytotoxicity of RPLEO in HepG2 and MCF-7 cancer cells. Following their contact with EO at different concentrations (50, 100, 200, and 400 µg/mL), the viability of both cancer cells decreased. When compared to the untreated control cells, the cellular proliferation of MCF-7 and HepG2 lines was significantly (*P* < 0.05) inhibited by 100 µg/mL of RPLEO, but less than the positive control (50 µg/mL of cisplatin). EO’s half-maximal inhibitory concentration (IC_50_) against HepG2 and MCF-7 cells was 112.34 ± 1.56 µg/mL and 126.34 ± 1.52, respectively (Fig. 5).

**Fig. 6:**
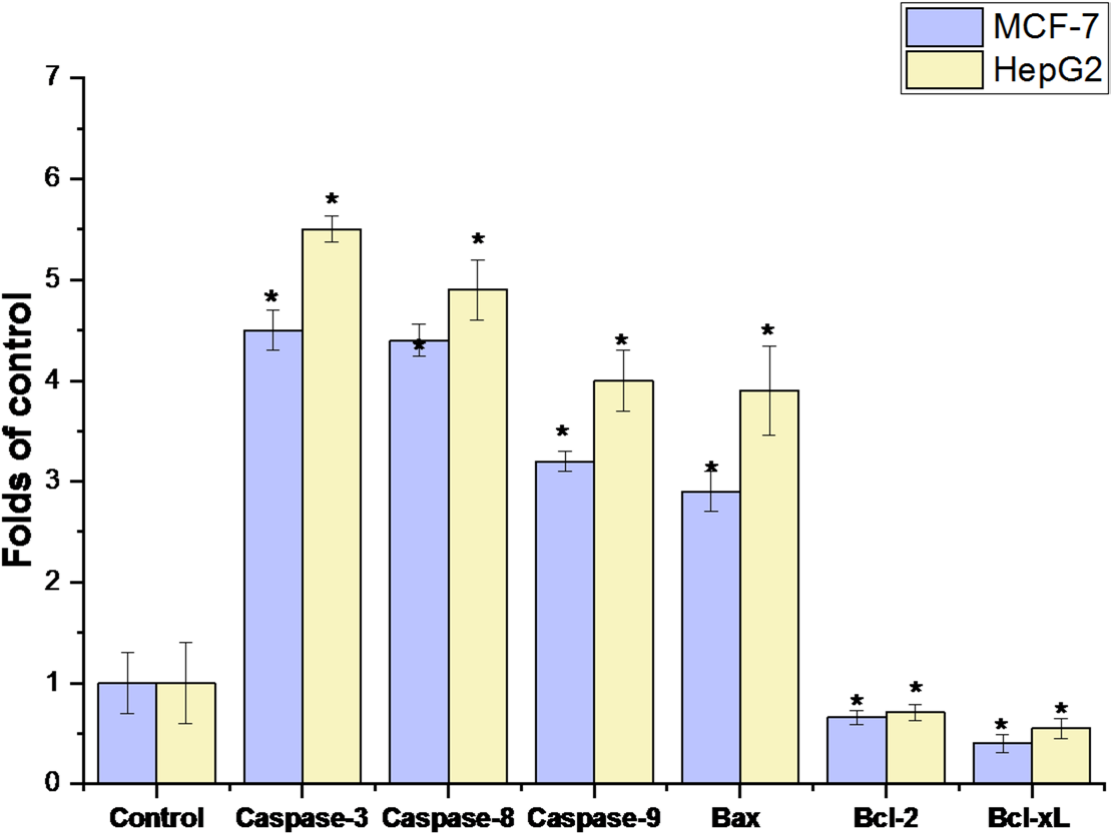
Effect of methanol extract of RPLEO on induction of apoptosis in human MCF-7 and HepG2 cells. The cells were treated without or with IC_50_ values of (120 µg/mL) of RPLEO for 48 h, collected, RNA isolated, and subjected to rRT-PCR. RPLEO significantly stimulates the activity of a percentage of the apoptotic population (*caspase-3*, *8*, *9*, and *Bax*) and anti-apoptotic genes (*Bcl-xL* and *Bcl-2*) compared to the control cells. The results are represented as the mean ± SD of three independent experiments (* = *P* < 0.05 compared to non-treated cells (control)).

The activity of apoptosis genes (caspase-3, 8, 9, and Bax) and anti-apoptotic genes (Bcl-xL and Bcl-2) was assessed by RT-PCR in the RPLEO. Caspases 3, 8, 9, and Bax activity were significantly higher in MCF-7 and HepG2 cells treated with 120 µg/mL of EO than in the control cells (*P* < 0.01). However, the expression of anti-apoptotic genes (Bcl-xL and Bcl-2) was lower than that of the untreated control (*P* < 0.05) (Fig. 6).

## Discussion

As no peer-reviewed study on RPLEO from Abha is available, we will extrapolate from adjacent *Resedaceae* in the vicinity. The hydro-distillation of RPLEO from Abha, Saudi Arabia, generated 4.75 ± 1.75% (v/w) essential oil, a significantly higher yield than other *Resedaceae* species. Steam distillation of Saudi *Reseda muricata* aerials produced approximately 0.13% oil (fresh basis)^11^. *Ochradenus arabicus* (*Resedaceae*) yields only 0.07–0.08% essential oil from aerial parts^6^. Hydro-distillation yields less than 1% essential oils from most *Resedaceae* plants, which is consistent with efficient extraction processes such as 20-day leaf drying and Clevenger equipment^9^. Comparable investigations on other *Resedaceae* members imply that RPLEO could be a useful bioactive resource, which warrants further exploration.

The GC-MS analysis of RPLEO from Abha, Saudi Arabia, revealed a complex profile rich in phenolic monoterpenoids (48.09%), particularly carvacrol (21.59%) as the predominant compound, along with trans-Dihydrocarvone (4.65), thymol (3.62%), followed by sesquiterpenoids (23.18%) such as Caryophyllene (3.62%), Cis-caryophyllene (2.06%), and Aromandendrene 2.37%). Other minor constituents included Phenylpropanes (13.12%) (6-tert-butyl-m-cresol and p-tert-butylanisole, cumenes (5.78%) (such as m-Cymene and 2-isopropyl-4-methylanisole), Oxanes (3.08%), and others. Other *Reseda* species have been studied, and their chemical profiles vary. *Reseda lutea* was found to be high in glucosinolates (e.g., benzyl glucosinolate) and flavonoids, which contribute to anti-inflammatory and antibacterial properties (Pagnotta et al., 2020). The methanolic extract of *Reseda muricata* contains alkaloids and flavonoids, which have antioxidant and antibacterial properties^12^. *Ochradenus arabicus* essential oils contain primarily isothiocyanates (47.5–84.3%) and terpenoids, which inhibit α-glucosidase (IC_50_ = 0.40 µg/mL)^6^. RPLEO’s high phenolic content suggests good antioxidant ability; however, direct bioactivity studies are required for validation.

A comparison of RPLEO’s chemical profile to previously investigated Resedaceae species indicates both similarities and differences. Ullah et al. [6] discovered that *Ochradenus arabicus* essential oils are predominantly composed of isothiocyanates (47.5–84.3%), including benzyl isothiocyanate and phenylpropyl isothiocyanate, which contrasts sharply with RPLEO’s phenolic monoterpenoid-rich profile. This discrepancy implies that the *Resedaceae* family exhibits significant chemotypic variation, which could be attributed to species-specific metabolic pathways or environmental influences. Al-Mazroa et al. [11] found carvacrol (12.8%) and thymol (4.2%) in *Reseda muricata* essential oil from Saudi Arabia, which is more akin to our findings for *R. pentagyna*, indicating that phenolic monoterpenoids may be unique to particular *Reseda* species. Al-Qurainy et al. [9] reported the existence of glucosinolate-derived chemicals in *R. pentagyna* methanolic extracts, but our GC-MS study of the essential oil fraction did not find these compounds, most likely due to changes in extraction methods and volatility. We confirmed the identities of compounds lacking CAS numbers discovered in our investigation (peaks 32, 41, 43, and 46) using mass spectral interpretation and comparison to available literature. Peak 32 (9-Isopropyl-1-methyl-2-methylene-5-oxatricyclo[5.4.0.0(3,8)]undecane) has already been identified in the essential oil of *Salvia* species [Rather et al., 2012]. Citrus essential oils include peak 41 (Geranyl-α-terpinene), while Apiaceae plant species contain peak 43 (Methyl octadeca-9-yn-11-trans-enoate) [Zidorn et al., 2005; Dugo et al., 2012]. Peak 46 (2-[4-methyl-6-(2,6,6-trimethylcyclohex-1-enyl)hexa-1,3,5-trienyl]cyclohex-1-en-1-carboxaldehyde) is a retinoid-like chemical previously found in Croton species [Salatino et al., 2007]. The discovery of these chemicals in RPLEO broadens our knowledge of the Resedaceae family’s chemical diversity.

Carvacrol, which is a strong antioxidant and antibacterial compound, is also consistent with studies on other *Resedaceae* members such as *Ochradenus arabicus*, where thymol (its structural analogue) is present and shown to possess the same biological activities^13^. Thymol has also been shown to have synergistic antibacterial activity since it is always mixed with carvacrol to enhance the membrane permeability of microbes and thus enhance activity against drug-resistant organisms. A study already demonstrated that Thymol suppresses the COX-2 and NF-κB signaling pathways, which can exhibit anti-inflammatory activity in arthritis and dermatitis models^14^. Another study has also demonstrated that Carvacrol has a wide spectrum of antibacterial activity against Gram-positive bacteria (e.g., *S. aureus*) and Gram-negative bacteria (e.g., *E. coli*), and antifungal activity against *Candida* species^15^. Its mechanism of action comprises disruption of the bacterial cell membranes and inhibition of biofilm. Carvacrol reduces pro-inflammatory cytokines (e.g., TNF-α, IL-6) and oxidative stress by free radical scavenging action^16^. Furthermore, it has been shown to induce apoptosis in cancer cells (e.g., colon and liver) by activating the mitochondrial pathway^17^^18^.

6-tert-Butyl-m-Cresol (11.48%) is an antioxidant with a comparable chemical composition to synthetic BHT^19^. It is a phenolic molecule that effectively removes reactive oxygen species (ROS) and suppresses lipid peroxidation. It is also known as cytoprotective, as it prevents oxidative damage to liver cells in vitro^19^. Trans-Dihydrocarvone biological activities have not been extensively studied; however, *Poiretia latifolia* leaf essential oils containing high levels of (+)-trans-Dihydrocarvone (77.8%) demonstrated some antimicrobial activity against *K. pneumoniae*, *S. epidermidis*,* Bacillus subtilis*,* S. aureus*, *E. coli*, and *Candida albicans*^20^. Worth noting is that two monoterpenoid cymene derivatives do occur, albeit at lower concentrations. The derivatives of cymene present in various plant essential oils possess a variety of pharmacological activities, including antioxidant, anti-inflammatory, analgesic, and antibacterial activities. They regulate cytokine levels and signaling pathways, thus providing neuroprotection and cardiovascular protection. P-cymene is also employed as an industrial intermediary in the synthesis of fragrances, insecticides, and antioxidant precursors^21^. RPLEO contains a high concentration of caryophyllene and its oxide, both of which have powerful anti-inflammatory properties. Caryophyllene, a CB2 receptor agonist, effectively decreases inflammation and pain in neuropathic and arthritic animals^22^. Notably, RPLEO lacks isothiocyanates, which are found in other *Resedaceae* (e.g., *Reseda lutea*’s glucosinolate-derived benzyl isothiocyanate), implying species-specific metabolisms^9^.

Folin-Ciocalteu assay determined 141.30 ± 1.72 mg GAE/g dry weight TPC, which is a sign of high bioactive phenolic chemicals. Similarly, a TFC of 88.34 ± 1.34 mg QE/g dry weight indicated high flavonoid content. These figures are intimidating compared to other medicinal plants in the region. There is little data on the TPC and TFC of the *Resedaceae* species. A study showed that the ethanolic extract of *Reseda lutea* had a TPC of 47.73 ± 0.32 µg/mg extract and a TFC of 84.43 ± 2.72 µg/mg extract^10^. Other studies on adjacent plant families provide an idea of the antioxidant potential of various plant species. *Rosa canina* L. Pseudo-Fruits showed decreased TPC concentration, ranging from 20 to 29 mg GAE/g ^23^. *Sidastrum micranthum* and *Wissadula periplocifolia* from the *Malvaceae* family have high antioxidant activity, with TPC and TFC of 177.44 ± 16.21 mg GAE/g and 260.46 ± 5.74 mg GAE/g, respectively^24^. RPLEO phenolic concentration is comparable to that of *Origanum vulgare*, an aromatic plant with a reported level of 120 to 150 mg/g GAE^25^. Because phenolics enhance membrane disruption in microorganisms, high TPC and TFC values suggest that RPLEO has the potential to reduce oxidative stress and promote antibacterial synergy.

The strong R² values for gallic acid (0.9976) and quercetin (0.9593) standard curves confirm the dependability of our quantitative analyses. An R² value close to 1.0 confirms the quality of the regression model used to calculate TPC and TFC, as it explains almost all the variance in absorbance based on standard concentration. These solid calibration curves reinforce confidence in the reported phenolic and flavonoid concentrations and assist the following interpretation of RPLEO’s bioactivities in connection with its phytochemical composition.

RPLEO showed strong antioxidant activity in a quantitative phytochemistry study. RPLEO antioxidant activity was determined by DPPH and ABTS radical scavenging assays. The assay showed dose-dependent activity of 80.13 ± 1.53 µg/mL versus DPPH and 92.97 ± 1.34 µg/mL versus ABTS. The values, though indicating strong antioxidant activity, were lower than the positive control ascorbic acid IC_50_ value of 30.61 ± 2.31 µg/mL. The antioxidant activity shown is due to the collective effort of several phenolic components found in the essential oil, including carvacrol and thymol, which are known for their hydrogen-donating capacity and subsequently induced a free radical scavenging activity. RPLEO is more antioxidant in character than other plants of the *Resedaceae* family. Water and ethanol extracts of *Reseda lutea* had weak antioxidant activities (DPPH IC_50_ = 231.0 ± 0.01 and 346.50 ± 0.03 µg/mL, respectively) due to their low content of phenolics^10^. *Ochradenus arabicus* essential oil has weak antioxidant activity (DPPH IC_50_ = 106.40 ± 0.19 µg/mL), but isothiocyanates, not phenolics 6 contribute its main activity. This comparison underlines the distinctive phytochemical composition of RPLEO and its potential as a source of natural antioxidants.

The antibacterial properties of RPLEO were thoroughly investigated utilizing disc diffusion assays, MIC, and MBC tests. Compared to the positive control, chloramphenicol (25 µg/mL), RPLEO showed considerable dose-dependent inhibition against both Gram-positive and Gram-negative bacteria. Gram-negative bacteria were more susceptible to RPLEO, with MICs ranging from 6.25 ± 0.00 to 12.50 ± 0.00 µg/mL, while Gram-positive bacteria had MICs between 12.50 ± 0.00 and 50.00 ± 0.00 µg/mL. RPLEO was exceptionally potent towards Gram-negative bacteria. *E. coli* was most susceptible with an MIC of 6.25 µg/mL and MBC of 12.50 µg/mL, followed by *K. pneumoniae* and *P. aeruginosa* (MIC = 12.50 µg/mL, MBC = 25.00 µg/mL). *E. faecalis* was the most sensitive Gram-positive bacterium (MIC = 12.50 µg/mL, MBC = 25.00 µg/mL), whereas *B. subtilis* was the most resistant (MIC = 50.00 µg/mL, MBC = 100.00 µg/mL). These findings suggest that RPLEO antibacterial fractions potentially possess a higher ability to penetrate or destroy Gram-negative bacterial cell walls. Greater potency against Gram-negative bacteria is due to RPLEO’s major phenolic constituent, carvacrol, whose ability has also been shown to disrupt bacterial membranes and augment antibiotic-like activity^15^. Relative to other *Resedaceae* species, while RPLEO was more active against Gram-negative bacteria, *Ochradenus arabicus* isothiocyanate-containing essential oils were less antibacterial but more antifungal in action (MIC > 50 µg/mL against the majority of the bacteria) (^6^. Also, the extracts of *Reseda lutea*, which are famous for glucosinolate-derived antimicrobials, are moderately active as antibacterial (MIC ~ 25–100 µg/mL), but RPLEO fares better compared to it against *E. coli* and *P. aeruginosa* (Al-Snafi, 2022). In comparison with other Mediterranean aromatic plants, carvacrol (21.59% in RPLEO in *Origanum syriacum* L. (Turkey) has MICs of 10–20 µg/mL against *P. aeruginosa*, which is similar to RPLEO’s findings^26^. RPLEO has competitive antibacterial action, especially against Gram-negative infections, compared to other regional essential oils.

RPLEO inhibited carbohydrate-splitting enzymes α-amylase (IC_50_ = 82.31 ± 1.28 µg/mL) and α-glucosidase (IC_50_ = 87.49 ± 1.19 µg/mL), and its potential as a natural antidiabetic agent is promising. *Ochradenus arabicus* is more potent against α-glucosidase inhibition (IC_50_ = 0.40 µg/mL) than RPLEO due to its greater content of isothiocyanates^6^. It does not inhibit α-amylase, unlike RPLEO, which inhibits both enzymes. Parallel to this, the Saudi date seed extracts inhibited α-glucosidase (IC_50_ = 51.71 ± 8.2 µg/mL), which was lower than that of RPLEO but rich in polyphenols such as flavonoids^27^. *Allium* spp. and *Nigella sativa* essential oils inhibited α-amylase more effectively than RPLEO^28^^29^. The observed suppression of these important carbohydrate-digesting enzymes may be related to the main phenolic chemicals (carvacrol, thymol) and other monoterpenoids, which can interact with the enzymes’ active sites, as seen in *Origanum vulgare*^30^. Furthermore, synergistic action of several RPLEO monoterpenoids may increase inhibition through modulation of enzyme-substrate binding, as shown in *Persicaria hydropiper* oils^31^. As the global diabetes pandemic grows, RPLEO and other *Resedaceae* species are an underutilized source of phytochemicals to act as adjuvants to antidiabetic treatment.

The cytotoxicity of RPLEO was tested on HepG2 (hepatocellular carcinoma) and MCF-7 (breast cancer) cell lines using the MTT assay. Cell viability was significantly reduced (*P* < 0.05) at concentrations of 100 µg/mL or higher than untreated controls. The IC_50_ values of MCF-7 and HepG2 cells were 112.34 ± 1.56 µg/mL and 126.34 ± 1.52 µg/mL, respectively, which are indicative of a slightly higher sensitivity of liver cancer cells towards the treatment with RPLEO. The stimulation of apoptosis, indicated by the upregulation of caspase-3, -8, -9, and Bax, aligns with the recognized propensity of key ingredients such as carvacrol and thymol to activate both intrinsic and extrinsic apoptotic pathways in cancer cells. The differential sensitivity of the two cell lines could be explained by differences in drug efflux mechanisms and cellular metabolism of the drugs targeting both cancers. On the genetic level, RPLEO-treated MCF-7 and HepG2 cells showed excellent modulation of pro-apoptotic (caspase-3, -8, -9, Bax) and anti-apoptotic (Bcl-2, Bcl-xL) genes. Caspase-3, -8, and − 9 overexpression shows the activation of both intrinsic (mitochondrial) and extrinsic (death receptor) apoptosis mechanisms. Caspase-9 induction implies mitochondrial dysfunction due to oxidative stress or DNA damage (Chen et al., 2022), whereas activation of caspase-8 indicates RPLEO constituents could interact with cell surface death receptors such as Fas^32^. The executioner caspase-3 reaffirms commitment to apoptosis irreversibly by DNA fragmentation and cellular shrinkage. At the same time, Bax overexpression destabilizes mitochondrial membrane potential, enabling cytochrome c release and amplifying caspase-9 activation^33^. These effects are reinforced through the intense downregulation of anti-apoptotic Bcl-2 and Bcl-xL, which in normal conditions stabilize mitochondria integrity and inhibit cytochrome c release. The resultant shift in the Bax/Bcl-2 ratio creates a permissive environment for apoptosis, reproducing mechanisms for other phenolic-rich essential oils.

Comparative studies have revealed RPLEO’s dual-pathway activation as a differentiating feature. The methanolic extract of *Ochradenus baccatus* (*Resedaceae*) showed excellent activity against A549 cancer cells (IC_50_ = 86.19 µg/mL)^34^. The ethanolic extract of *Ochradenus arabicus* (*Resedaceae*) exhibited poor cytotoxicity (IC_50_ > 562 µg/mL) against MCF-7 ^35^, while its biosynthesized silver nanoparticles increased the cytotoxicity rate (IC_50_ = 100 µg/mL) and initiated ROS generation^36^, in contrast to RPLEO’s moderate effect. *Ochradenus arabicus* causes apoptosis mostly through p53 overexpression, with no significant caspase-8 involvement, demonstrating RPLEO’s broad molecular range^9^. Similarly, *Artemisia arborescens* essential oils promote apoptosis in prostate cancer cells by inhibiting STAT-3 and producing ROS, although without the accompanying extrinsic pathway involvement described in RPLEO^37^. In the same context, the cytotoxicity of *Reseda lutea* was greater on human melanoma (A375) and fibroblast (MRC5) cell lines, and apoptosis was caused by benzyl isothiocyanate^38^. The activity of *Reseda lutea*, as compared with the phenolic-controlled RPLEO, is isothiocyanate-controlled and exhibits the genus’s metabolic adaptability. De et al. (2022) found that *Dillenia pentagyna*, a Mediterranean herb, showed lower cytotoxicity (IC_50_ > 350 µg/mL) on A594 cells than RPLEO^39^. Furthermore, root extracts of *Leonurus sibiricus* (*Lamiaceae*) had high cytotoxicity (IC_50_ < 50 µg/mL) against leukemia (CCRF-CEM, K-562) through mitochondrial apoptosis^40^, outperforming RPLEO against hematological malignancies. RPLEO cytotoxicity, however mild compared to other *Resedaceae* and natural plants, validates its phenolic-mediated anticancer action. RPLEO’s phenolic components, particularly carvacrol and thymol, are likely to be responsible for these effects, as both chemicals have been shown to affect mitochondrial function and enhance death receptor signaling in other systems.

This study brings new information about the biological features of RPLEO, such as its outstanding antioxidant capacity, record levels of phenolic and flavonoid content, and high activity of radical scavenging. The current study is the first to demonstrate inhibition of α-amylase and α-glucosidase, indicating potential for antidiabetic usage. The study also demonstrates, for the first time, antibacterial efficacy against Gram-negative infections with bacterial species-specific sensitivity. It also has anticancer properties against HepG2 and MCF-7 cell lines. This study provides the first evidence that *R. pentagyna* essential oil is a distinct, phenol-rich chemotype with multifunctional biological activities, filling a critical knowledge gap in the phytochemistry of Saudi endemic flora and the *Resedaceae* family.

While this work gives a basic overview of RPLEO, some limitations should be noted. The bioactivities given are based on in vitro experiments, which, while useful, do not entirely reproduce the complexity of in vivo physiological systems. The phytochemical quantification is based on GC-MS peak area normalization, which yields a reliable relative composition but is semi-quantitative and sensitive to compound-specific response variables. Future research should use methodologies like Gas Chromatography with Flame Ionization Detection (GC-FID) with real standards for definitive quantification. Furthermore, the observed benefits are assigned to the entire essential oil; the individual contributions and potential synergy or antagonism among its 53 identified constituents remain to be determined by compound isolation investigations. The absence of comparative evaluation using normal cell lines (such as MCF-10 A normal breast epithelial cells, normal hepatocytes, or peripheral blood mononuclear cells) represents a limitation and prevents definitive conclusions regarding cytotoxic selectivity.

## Materials and methods

### Plant material and extraction

*R. pentagyna* was collected from Abha, Saudi Arabia. The collection of plant materials was conducted following the guidelines of the International Union for Conservation of Nature (IUCN) policies, research involving species at risk of extinction, and the Convention on International Trade in Endangered Species of Wild Fauna and Flora (CITES). We affirm that the *R. pentagyna* is not subject to trade restrictions under CITES and is not designated as protected or endangered under either national laws or the IUCN Red List. We further confirm that the collection and use of plant material in this study complied with all relevant institutional and national guidelines and legislation. The plant was identified at King Saud University by Professor Dr. Mohammed Fasil. The voucher specimen was deposited in the College of Science’s Botany and Microbiology Herbarium at King Saud University (KSU NO-37452). The *R. pentagyna* leaves were thoroughly cleansed under running water to remove any surface impurities. They were then allowed to dry for 20 days at room temperature to ensure consistent drying of *R. pentagyna.* The air-dried *R. pentagyna* leaves were next ground into a powder in a space with sufficient ventilation. As previously stated, RPLEO were extracted by hydrodistillation, 100 g of dried *R. pentagyna* leaves were distilled for three hours with 1,000 mL of distilled water using a Clevenger-style device. Following collection and drying on anhydrous sodium sulphate, the extracted RPLEO was stored at -4 °C in sealed glass vials until it was required.

### Identification of bioactive compounds

A single quadrupole mass analyzer capillary HP-5MS UI (Ultra Inert), length: 30 m, i.d.: 0.25 mm, film thickness: 0.25 μm, stationary phase: 5% phenyl, methylpolysiloxane (low polar) from Agilent Technologies (Santa Clara, CA, USA), was used in the GC-MS system Agilent 7890B coupled to Agilent 5977 A MSD. The temperature was set at 7.5 °C/min constant, 280 °C for 15 min, 250 °C for 1 min, and 50 °C for 3 min. The mass range is 50-1000 Da g/mol, the acquisition scan type was full scan, the liquid delay was 4 min, and the scan speed is 1.56 u/sec. A steady flow rate of 1 mL/ minute was maintained for the carrier gas (helium). The GC-MS was used in EI ionization mode with an ionization voltage of 70 eV to gather the results. The injector and detector were set to operate at 250 °C and 300 °C, respectively. Willey and the mass spectral database of the National Institute of Standards and Technology (NIST) served as the search libraries. Compounds with a matching factor above 90% were identified by comparing the components with those in the NIST computer libraries linked to the GC-MS apparatus. The relative percentages of identified substances were computed using peak area normalization from the GC-MS total ion chromatogram (TIC). It is known that this method produces a semi-quantitative approximation because the MS response factor varies among compound classes.

Compounds with a matching factor above 90% were identified by comparing the components with those in the NIST computer libraries linked to the GC-MS apparatus. For compounds lacking CAS numbers or with ambiguous library matches, identification was further verified by manual interpretation of mass spectral fragmentation patterns and comparison with published literature reporting these compounds in other plant species. The relative percentages of identified substances were computed using peak area normalization from the GC-MS total ion chromatogram (TIC). It is known that this method produces a semi-quantitative approximation because the MS response factor varies among compound classes.

### Determination of TPC and TFC

The TPC was determined using the Folin–Ciocalteu method, as described in ^41^. A solution of 0.1 mL of plant extract (1 mg/mL) and 3 mL of distilled water was mixed with 2 mL of 20% NaHCO_3_ and 0.5 mL of Folin-Ciocalteu reagent. After thoroughly mixing the mixture, it was incubated for 20 min at 45 °C. The optical density (OD) was measured at 725 nm using a spectrophotometer (U2001 UV-vis Spectrophotometer, Hitachi, Japan). GAE/extract mg was used to express the results.

AlCl_3_ was used to determine the TFC, as explored in reference^42^. Briefly, 500 µL of each extract was combined with 1 mL of AlCl_3_ that had been dissolved in ethanol. After that, 3 mL of sodium acetate solution (50 mg/L) was added to the mixture. Two drops of acetic acid were then added. The resulting solution was incubated for 30 min in a dark environment at 23 °C. The OD was measured with a spectrophotometer (U2001 UV-vis Spectrophotometer, Hitachi, Japan) at a wavelength of 415 nm. Based on the QAE standard, a standard curve was created to compute the TPC. The results showed up as mg QAE/g of extract.

Calibration curves were evaluated for linearity by obtaining the coefficient of determination (R²) by linear regression. The calibration model accurately quantifies phenolic and flavonoid content in samples because of a strong linear relationship between standard concentration and absorbance, as indicated by R² values close to 1.0.

### Antioxidant activity

#### DPPH assay

Using the previously mentioned methodology^43^, the DPPH radical scavenging assay was used to evaluate the RPLEO capacities to react with DPPH radicals. The extracts were prepared at five concentrations (50, 100, 200, 400, and 800 µg/mL). Approximately 0.5 mL of the extract was mixed with 0.375 mL of methanol and a DPPH solution (2 mL, 0.08 mM) for each concentration. The reaction mixture was then placed in a dark environment and incubated for 30 min. After incubation, the OD of the mixture was measured at 517 nm using a spectrophotometer (U2001 UV–vis Spectrophotometer, Hitachi, Japan). Ascorbic acid was used as a positive control at known concentrations (400 µg/mL). The proportion of DPPH scavenging activity (%) was determined using the following equation: DPPH radical scavenging activity [%] = [(Ac - A)/Ac] × 100, where Ac denotes the absorbance of the control, and A denotes the absorbance of the sample. The half-maximal inhibitory concentration (IC_50_) value was computed using GraphPad Prism software (version 5.0, La Jolla, CA, USA). The IC_50_ of DPPH scavenging activity was determined graphically using the line equation obtained from the % value of the antioxidant activity and the concentration value plotted on a graph, where the concentration value is on the *X*-axis, and the % activity is on the *Y*-axis^44^.

#### ABTS assay

The ABTS radical scavenging assay was conducted using the methodology outlined in a previous study^45^. As the positive control, ascorbic acid (200 µl, 400 µg/mL) was used. The ABTS solution (192 mg/50 mL) and the K_2_S_2_O_8_ solution (140 mM) were first combined to perform the test. The reaction mixture was therefore allowed to sit at room temperature for around twelve hours in the dark. Additionally, the ABTS solution was mixed with methanol to provide an OD of 0.70 ± 0.02 at 734 nm. Additionally, 3 mL of diluted ABTS was thoroughly combined with 50 µL of each extract concentration. Additionally, the mixture was incubated in a dark environment for six minutes. A spectrophotometer (U2001 UV–vis Spectrophotometer, Hitachi, Japan) was then used to detect the OD at 734 nm. As previously mentioned, the results are displayed as IC_50_ and ABTS% values.

### Antibacterial activity

#### Disc diffusion assay

The antimicrobial properties of RPLEO were evaluated against three Gram-negative strains: *E. coli* (ATCC-25922), *K. pneumoniae* (ATCC − 13883), and *P. aeruginosa* (ATCC-27853), and three Gram-positive strains: *S. aureus* (ATCC-29213), *E. faecalis* (ATCC-29212), and *B. subtilis (*ATCC − 21332), as previously mentioned^46^. These strains were chosen because they represent clinically relevant pathogens with differing cell wall structures, allowing a comprehensive evaluation of the antibacterial spectrum of RPLEO. These bacteria were swabbed on Mueller Hinton Agar (MHA) medium at 37 °C for 24 h (0.1 mL, 1 × 10^6^ CFU/mL saline). The next step included incubating the petri dishes at 37 °C for 24 h. Hole-punching with a 5 mm diameter cork-borer was used to create wells at equal surface intervals. The RPLEO (100, 200, 400, and 800 µg/mL; 100 µL/well) were mixed with the resulting wells in triplicate and placed on inoculated Petri plates. Chloramphenicol (25 µg/mL) was used as a positive control, whereas Muller-Hinton Broth (MHB) was used as the negative control. After incubation, ZoI was assessed in millimeters (mm)^47^.

### MIC and MBC

The broth dilution procedure used the 2,3,5-triphenyl tetrazolium chloride (TTC) method to determine the RPLEO’s MIC and MBC^48^. MHB (100 µl) was mixed with varying amounts of RPLEO (1.95 to 800 µg/mL) on a 96-well plate. After adding 10 µl of bacterial solution, each well had a total bacterium count of 5 × 10^6^ colony-forming units (CFU)/mL. The positive control included 25 µg/mL of chloramphenicol, whereas the negative control was MHA. The plates were examined visually for the presence of bacteria after being incubated for 24 h at 37 °C. Following the addition of 20 µL TTC working solution in PBS (2 mg/mL) to each well, the mixture was incubated for 20 min at 37 °C. Colorless solution wells were considered negative for bacterial growth, but pink solution wells that mirrored the positive control were considered positive. MBC, the lowest concentration indicating subculture, and MIC, the lowest concentration inhibiting bacterial growth, were identified^49^.

### Antidiabetic activity

#### In vitro α-amylase inhibition assay

As previously reported, the 3,5-dinitrosalicylic acid (DNSA) assay was employed to determine whether α-amylase activity was inhibited^50^. In brief, RPLEO was diluted with the buffer (0.02 M Na_2_HPO_4_/NaH_2_PO_4_; 0.006 M NaCl; pH 6.9) to achieve concentrations between 50 and 1000 µg/mL. After mixing 200 µl of each extract with 200 µL of the Molychem α-amylase solution (2 units/mL), the mixture was incubated for 10 min at 37 °C. After that, 200 µL of the 1% starch solution (w/v) was added to each tube, and the tubes were kept at 37 °C for three minutes. After adding 200 µL of DNSA reagent (12 g of sodium potassium tartrate tetrahydrate in 8.0 mL of 2 M NaOH and 20 mL of 96 mM 3,5-DNSA solution) to stop the reaction, it was heated for 10 min at 85 °C in a water bath. The positive control was 400 µg/mL of acarbose (Bayer) in 100 µL. After letting the sample cool to room temperature and diluting it with 5 mL of distilled water, the OD at 540 nm was measured using a UV-visible spectrophotometer (U2001 UV-vis Spectrophotometer, Hitachi). The α-amylase inhibition was calculated and expressed as a percentage of inhibition, and IC_50_ values were ascertained using GraphPad Prism software (version 5.0, La Jolla, CA, USA).

#### In vitro α-glucosidase inhibition assay

The α-glucosidase inhibitory activity was evaluated using yeast α-glucosidase and p-nitrophenyl-α-D-glucopyranoside (pNPG), as was previously described^51^. To achieve a final concentration of 0.5 to 5.0 mg/mL, the extracts (100 µl, 20 mg/mL) or 25–400 µg/mL of acarbose (as a positive control) were added to 50 µL of α-glucosidase (1 U/mL) prepared in 0.1 M phosphate buffer (pH 6.9) and 250 µL of 0.1 M phosphate buffer.

The mixture was incubated at 37 °C for 20 min. After adding 10 µL of 10 mM pNPG made in a 0.1 M phosphate buffer (pH 6.9), the mixture was incubated at 37 °C for an hour. Using a spectrophotometer (U2001 UV–vis Spectrophotometer, Hitachi, Japan), the OD was measured at 405 nm following the addition of 650 µL of 1 M sodium carbonate to stop the reactions. The results were represented as a percentage of the enzyme activity inhibition (%), and IC_50_ values were calculated using GraphPad Prism.

### Cell culture and cytotoxicity assays

This study examined the toxic characteristics of RPLEO utilizing the most widely used cancer cell lines for assessing the cytotoxicity of plant extracts in vitro: Michigan Cancer Foundation-7 (MCF-7) (ATCC HTB-22) and Hepatocellular carcinoma cell (HepG2) (ATCC HB-8065)^52^^53^. The cells were cultivated in Dulbecco’s Modified Eagle Medium (DMEM), which was enhanced with fetal calf serum (FCS) and 1% penicillin-streptomycin. The cells were cultivated in a humidified atmosphere with 5% CO_2_ at 37 °C. MTT assay was used to assess cell viability. The MTT assay’s basic idea is that mitochondria convert yellowish MTT into purple formazan granules^54^. 100 µL of DMEM (100 mL) was added to each well of the 96-well microplate for inoculation. A completely formed monolayer sheet was created after incubating the microplate for 24 h at 37 °C, 95% humidity, and 5% CO_2_. Using a growth medium, 0.1% dimethyl sulfoxide (DMSO)-solubilized extracts were serially diluted at 50–400 µg/mL. Following the extract treatment, the cells were incubated at 37 °C with 5% CO2 for 24 h. The positive control was 30 µg/mL of cisplatin. Negative control cells were incubated without the addition of RPLEO. Each well was then filled with 10 µL of MTT solution. For mixing, a shaker (MPS-1, Biosan, London, UK) was utilized, and it was operated at 150 rpm for five minutes. For four hours, the incubation process was maintained. The MTT assay’s metabolic byproduct, formazan, was reconstituted in 100 µL of DMSO and forcefully shaken for five minutes at 150 rpm. An ELX-808 microplate reader (BioTek Laboratories, LL, Shoreline, W.A., USA) was used to measure the optical density at 570 nm. A background reference wavelength of 620 nm was used to adjust the data. The findings were shown as a percentage of cell viability (%), and GraphPad Prism (version 5.0, La Jolla, CA, USA) was used to get the IC_50_ values after the mean value ± SD was considered for data processing^55^.

#### RT-PCR assay of apoptosis genes

The expression of apoptotic (caspase-3, 8, 9, and Bax) and anti-apoptotic (Bcl-xL and Bcl-2) genes was evaluated using RT-PCR. Two thousand cells of MCF-7 and HepG2 were gathered and placed in six-well plates containing 100 µg/mL of the RPLEO. The pellet was subjected to RNA extraction using a RNeasy kit (Qiagen, Hilden, Germany) following centrifugation at 500 g for 5 min at 4 °C. The supernatant was then extracted. This RNA was used as the RT-PCR template. A 7500 Fast real-time PCR (7500 Fast; Applied Biosystems, Foster City, CA, USA) was used to conduct the RT-PCR assays as described in our recent study^56^.

### Statistical analysis

The results were analyzed using GraphPad PRISM (version 5.0, La Jolla, CA, USA). Three separate experiments were conducted in triplicate, and the mean value ± standard deviation (SD) was reported. The means of two independent groups were compared using Student’s unpaired t-test. The groups were compared using the Mann-Whitney U test for non-Gaussian variables. For antibacterial assay, statistical differences among groups were analyzed using one-way analysis of variance (ANOVA), followed by Dunnett’s post-hoc test, with all treatment groups compared to the positive control. Differences were considered significant when the *p*-value was < 0.05.

## Conclusions

This innovative study provides the first thorough phytochemical and pharmacological characterization of essential oil derived from the Saudi indigenous plant RPLEO. The important findings show that RPLEO is a high-yielding oil with a distinct phenolic-rich content, characterized by carvacrol as the major component along with appreciable amounts of thymol and other bioactive components. The chemical profile of this substance supports its multi-target bioactivities, including potent antioxidant capacity due to high phenolic content, strong antibacterial effects against Gram-negative pathogens, inhibition of diabetes-related enzymes (α-amylase and α-glucosidase), and cytotoxic activity against liver and breast cancer cell lines via apoptosis induction. These findings place RPLEO as a possible source of bioactive natural chemicals. To realize this potential, future research should focus on isolating pure active principles, validating efficacy in animal models, and investigating synergistic interactions with conventional therapies.

## Data Availability

The datasets used and/or analysed during the current study are available from the corresponding author on reasonable request.

## References

[1] Kashyap, A., Sarma, A., Das, B. K. & Goswami, A. K. Rational design of natural products for drug discovery. computational methods ra*tion. Drug Design* 285–309 (2025).

[2] Javed, A., Hashmi, M. S., Javaid, U. & Amjad, R. Classification and therapeutic applications of plant secondary metabolites. *Pharmacognosy and Phytochemistry: Principles, Techniques, and Clinical Applications* 189–206 (2025).

[3] Martín-Bravo, S. et al. Molecular systematics and biogeography of Resedaceae based on ITS and trnL-F sequences. *Mol. Phylogenet. Evol.* **44**, 1105–1120 (2007). 17300965 10.1016/j.ympev.2006.12.016

[4] Punt, W. & Marks, A. R. *Rev. Palaeobot. Palynol.* **88**, 47–59 (1995).

[5] Mitsiogianni, M. et al. The role of isothiocyanates as cancer chemo-preventive, chemo-therapeutic and anti-melanoma agents. *Antioxidants* **8**, 106 (2019). 31003534 10.3390/antiox8040106 PMC6523696

[6] Ullah, O. et al. Bilal S.Aroma profile and biological effects of Ochradenus arabicus essential oils: A comparative study of stem, flowers, and leaves. *Molecules* **27**, 5197 (2022). 36014440 10.3390/molecules27165197 PMC9414473

[7] Ali, M. A. et al. Status of Reseda pentagyna Abdallah & AG Miller (Resedaceae) inferred from combined nuclear ribosomal and chloroplast sequence data. *Bangladesh J. Plant. Taxonomy*. **20**, 233–238 (2013).

[8] Cilden, E. & Yildirimli, Ş. Taxonomic revision of the genus Reseda L.(Resedaceae) in Turkey. (2020).

[9] Al-Qurainy, F. et al. Estimation of genome size in the endemic species Reseda pentagyna and the locally rare species Reseda lutea using comparative analyses of flow cytometry and k-mer approaches. *Plants* **10**, 1362 (2021). 34371565 10.3390/plants10071362 PMC8309327

[10] Kiziltaş, H. Comprehensive evaluation of *Reseda lutea* L.(Wild Mignonette) and 7 isolated flavonol glycosides: determination of antioxidant activity, anti-Alzheimer, antidiabetic and cytotoxic effects with in vitro and in silico methods. *Turk. J. Chem.* **46**, 1185–1198 (2022). 37538778 10.55730/1300-0527.3426 PMC10395699

[11] Al-Mazroa, S., Al-Wahaibi, L., Mousa, A. & Al-Khathlan, H. Essential oil of some seasonal flowering plants grown in Saudi Arabia. *Arab. J. Chem.* **8**, 212–217 (2015).

[12] Ullah, R. & Alqahtani, A. S. GC-MS analysis, heavy metals, biological, and toxicological evaluation of *Reseda muricata* and Marrubium vulgare methanol extracts. *Evidence-Based Complementary and Alternative Medicine* 2284328 (2022). 10.1155/2022/2284328 PMC8959963 35356243

[13] Chroho, M., Rouphael, Y., Petropoulos, S. A. & Bouissane, L. Carvacrol and thymol content affects the antioxidant and antibacterial activity of Origanum compactum and Thymus zygis essential oils. *Antibiotics* **13**, 139 (2024). 38391524 10.3390/antibiotics13020139 PMC10885931

[14] Islam, M. T. et al. El-Nashar HA.Anti-inflammatory effects of thymol: an emphasis on the molecular interactions through in vivo approach and molecular dynamic simulations. *Front. Chem.* **12**, 1376783 (2024). 38983677 10.3389/fchem.2024.1376783 PMC11231963

[15] Mączka, W., Twardawska, M. & Grabarczyk, M. Wińska K.Carvacrol—A natural phenolic compound with antimicrobial properties. *Antibiotics* **12**, 824 (2023). 37237727 10.3390/antibiotics12050824 PMC10215463

[16] Potra Cicalău, G. I. et al. Ganea M.Assessing the antioxidant benefits of topical carvacrol and magnolol periodontal hydrogel therapy in periodontitis associated with diabetes in wistar rats. *Dentistry J.* **11**, 284 (2023). 10.3390/dj11120284 PMC10742747 38132422

[17] Fan, K. et al. Carvacrol inhibits proliferation and induces apoptosis in human colon cancer cells. *Anti-cancer drugs*. **26**, 813–823 (2015). 26214321 10.1097/CAD.0000000000000263

[18] Sampaio, L. A., Pina, L. T. S., Serafini, M. R. & Tavares, D. S. Guimaraes AG.Antitumor effects of carvacrol and thymol: a systematic review. *Front. Pharmacol.* **12**, 702487 (2021). 34305611 10.3389/fphar.2021.702487 PMC8293693

[19] Wang, W., Kannan, P., Xue, J. & Kannan, K. Synthetic phenolic antioxidants, including butylated hydroxytoluene (BHT), in resin-based dental sealants. *Environ. Res.* **151**, 339–343 (2016). 27522571 10.1016/j.envres.2016.07.042

[20] Porto, C. et al. Morel AF.(R)-(-)-carvone and (1R, 4R)-trans-(+)-dihydrocarvone from poiretia latifolia vogel. *J. Braz. Chem. Soc.* **21**, 782–786 (2010).

[21] Abbas, M. et al. Antimicrobial properties and therapeutic potential of bioactive compounds in nigella sativa: a Review. *Molecules* **29**, 4914 (2024). 39459282 10.3390/molecules29204914 PMC11510594

[22] Aly, E., Khajah, M. A. & Masocha, W. β-Caryophyllene, a CB2-receptor-selective phytocannabinoid, suppresses mechanical allodynia in a mouse model of antiretroviral-induced neuropathic pain. *Molecules* **25**, 106 (2019). 31892132 10.3390/molecules25010106 PMC6983198

[23] Nicolescu, A. et al. Rocchetti G.Optimized ultrasound-assisted enzymatic extraction of phenolic compounds from Rosa canina L. pseudo-fruits (rosehip) and their biological activity. *Antioxidants* **11**, 1123 (2022). 35740020 10.3390/antiox11061123 PMC9220760

[24] de Oliveira, A. M. F. et al. Assis TS.Total phenolic content and antioxidant activity of some Malvaceae family species. *Antioxidants* **1**, 33–43 (2012). 26787614 10.3390/antiox1010033 PMC4665395

[25] Morshedloo, M. R., Salami, S. A., Nazeri, V., Maggi, F. & Craker, L. Essential oil profile of oregano (*Origanum vulgare* L.) populations grown under similar soil and climate conditions. *Ind. Crops Prod.* **119**, 183–190 (2018).

[26] Alma, M. H., Mavi, A., Yildirim, A., Digrak, M. & Hirata, T. Screening chemical composition and in vitro antioxidant and antimicrobial activities of the essential oils from Origanum syriacum L. growing in Turkey. *Biol. Pharm. Bull.* **26**, 1725–1729 (2003). 14646179 10.1248/bpb.26.1725

[27] Khan, S. A., Al Kiyumi, A. R., Al Sheidi, M. S., Al Khusaibi, T. S. & Al Shehhi, N. M. Alam T.In vitro inhibitory effects on α-glucosidase and α-amylase level and antioxidant potential of seeds of Phoenix dactylifera L. *Asian Pac. J. Trop. Biomed.* **6**, 322–329 (2016).

[28] Ahmed, M. U., Ibrahim, A. & Dahiru, N. J. Mohammed HuS.Alpha amylase inhibitory potential and mode of inhibition of oils from *Allium sativum* (Garlic) and *Allium cepa* (Onion). *Clin. Med. Insights: Endocrinol. Diabetes*. **13**, 1179551420963106 (2020). 33088187 10.1177/1179551420963106 PMC7545766

[29] Dalli, M. et al. In vitro α-amylase and hemoglobin glycation inhibitory potential of Nigella sativa essential oil, and molecular docking studies of its principal components. *Front. Pharmacol.* **13**, 1036129 (2022). 36339531 10.3389/fphar.2022.1036129 PMC9631318

[30] Salazar, M. O., Osella, M. I., Arcusin, D. E., Lescano, L. E. & Furlan, R. L. New α-glucosidase inhibitors from a chemically engineered essential oil of Origanum vulgare L. *Ind. Crops Prod.* **156**, 112855 (2020).

[31] MahnashiMH et al. Phytochemical Analysis, α-Glucosidase and Amylase Inhibitory, and Molecular Docking Studies on Persicaria hydropiper L. Leaves Essential Oils. *Evidence-Based Complement. Altern. Med.* **7924171** (2022). 10.1155/2022/7924171 PMC8791729 35096118

[32] Tummers, B. & Green, D. R. Caspase-8: regulating life and death. *Immunol. Rev.* **277**, 76–89 (2017). 28462525 10.1111/imr.12541 PMC5417704

[33] Saddam, M. et al. Emerging biomarkers and potential therapeutics of the BCL-2 protein family: the apoptotic and anti-apoptotic context. *Egypt. J. Med. Hum. Genet.* **25**, 12 (2024).

[34] Khojali, W. M. et al. Alshammari RAR.Chemical composition, antibacterial activity and in vitro anticancer evaluation of Ochradenus baccatus methanolic extract. *Medicina* **59**, 546 (2023). 36984547 10.3390/medicina59030546 PMC10054464

[35] Ali, M. A. et al. Assessment of biological activity and UPLC–MS based chromatographic profiling of ethanolic extract of *Ochradenus arabicus*. *Saudi J. Biol. Sci.* **23**, 229–236 (2016). 26981004 10.1016/j.sjbs.2015.02.010 PMC4778516

[36] Al-kawmani, A. A. et al. Apoptosis-inducing potential of biosynthesized silver nanoparticles in breast cancer cells. *J. King Saud University-Science*. **32**, 2480–2488 (2020).

[37] Russo, A., Graziano, A., Bruno, M., Cardile, V. & Rigano, D. Apoptosis induction of essential oils from *Artemisia arborescens* L. in human prostate cancer cells. *J. Ethnopharmacol.* **303**, 115929 (2023). 36379416 10.1016/j.jep.2022.115929

[38] Radulović, N. S., Zlatković, D. B., Ilić-Tomić, T., Senerović, L. & Nikodinovic-Runic, J. Cytotoxic effect of *Reseda lutea* L.: A case of forgotten remedy. *J. Ethnopharmacol.* **153**, 125–132 (2014). 24509155 10.1016/j.jep.2014.01.034

[39] De, D., Chowdhury, P. & Panda, S. K. Ghosh U.Leaf extract and active fractions of Dillenia pentagyna Roxb. reduce in vitro human cancer cell migration via NF-κB pathway. *Integr. Cancer Ther.* **21**, 15347354221128832 (2022). 36419372 10.1177/15347354221128832 PMC9703490

[40] Sitarek, P., Synowiec, E., Kowalczyk, T. & Śliwiński, T. & Skała E.An in vitro estimation of the cytotoxicity and genotoxicity of root extract from *Leonurus sibiricus* L. overexpressing AtPAP1 against different cancer cell lines. Molecules. 23, (2049). (2018). 10.3390/molecules23082049 PMC6222913 30115821

[41] Wolfe, K. L. & Liu, R. H. Apple peels as a value-added food ingredient. *J. Agric. Food Chem.* **51**, 1676–1683 (2003). 12617604 10.1021/jf025916z

[42] Ordonez, A., Gomez, J. & Vattuone, M. Antioxidant activities of Sechium edule (Jacq.) Swartz extracts. *Food Chem.* **97**, 452–458 (2006).

[43] Tian, M. et al. Phytochemical analysis, antioxidant, antibacterial, cytotoxic, and enzyme inhibitory activities of *Hedychium flavum* rhizome. *Front. Pharmacol.* **11**, 572659 (2020). 33041813 10.3389/fphar.2020.572659 PMC7528636

[44] Reviana, R. et al. Analysis of antioxidant activity on cocktail honey products as female pre-conception supplements. *Gac. Sanit.* **35**, S202–S205 (2021). 34929812 10.1016/j.gaceta.2021.10.021

[45] Yu, X., Zhao, M., Liu, F., Zeng, S. & Hu, J. Antioxidants in volatile Maillard reaction products: Identification and interaction. *LWT-Food Sci. Technol.* **53**, 22–28 (2013).

[46] Salem, N. et al. Variation in chemical composition of *Eucalyptus globulus* essential oil under phenological stages and evidence synergism with antimicrobial standards. *Ind. Crops Prod.* **124**, 115–125 (2018).

[47] Singh, P. et al. Extracellular synthesis of silver and gold nanoparticles by Sporosarcina koreensis DC4 and their biological applications. *Enzym. Microb. Technol.* **86**, 75–83 (2016). 10.1016/j.enzmictec.2016.02.005 26992796

[48] Basri, D. F. & Sandra, V. Synergistic interaction of methanol extract from Canarium odontophyllum Miq. Leaf in combination with oxacillin against methicillin-resistant Staphylococcus aureus (MRSA) ATCC 33591. *Int. J. Microbiol.* (2016). 10.1155/2016/5249534 PMC4781960 27006659

[49] Aljeldah, M. M., Yassin, M. T., Mostafa, A. A. F. & Aboul-Soud, M. A. Synergistic antibacterial potential of greenly synthesized silver nanoparticles with fosfomycin against some nosocomial bacterial pathogens. *Infection Drug Resistance* 125–142 (2022). 10.2147/IDR.S394600 PMC9831080 36636381

[50] Wickramaratne, M. N., Punchihewa, J. & Wickramaratne, D. In-vitro alpha amylase inhibitory activity of the leaf extracts of *Adenanthera pavonina*. *BMC Complement. Altern. Med.* **16**, 1–5 (2016). 27846876 10.1186/s12906-016-1452-y PMC5109804

[51] Kim, Y-M., Wang, M-H. & Rhee H-I.A novel α-glucosidase inhibitor from pine bark. *Carbohydr. Res.* **339**, 715–717 (2004). 15013410 10.1016/j.carres.2003.11.005

[52] Mosmann, T. Rapid colorimetric assay for cellular growth and survival: application to proliferation and cytotoxicity assays. *J. Immunol. Methods*. **65**, 55–63 (1983). 6606682 10.1016/0022-1759(83)90303-4

[53] Alkhudhayri, A. A., Wahab, R., Siddiqui, M. A. & Ahmad, J. Selenium nanoparticles induce cytotoxicity and apoptosis in human breast cancer (MCF-7) and liver (HEPG2) cell lines. *Nanosci. Nanatechnol. Lett.* **12**, 324–330 (2020).

[54] Ghasemi, M., Turnbull, T., Sebastian, S. & Kempson, I. The MTT assay: utility, limitations, pitfalls, and interpretation in bulk and single-cell analysis. *Int. J. Mol. Sci.* **22**, 12827 (2021). 34884632 10.3390/ijms222312827 PMC8657538

[55] Al-Dhabi, N. A. & Valan Arasu, M. Quantification of phytochemicals from commercial Spirulina products and their antioxidant activities. *Evidence-Based Complementary and Alternative Medicine* (2016). 10.1155/2016/7631864 PMC4737012 26933442

[56] Aziz, I. M. et al. Chemical composition, antioxidant, anticancer, and antibacterial activities of roots and seeds of *Ammi visnaga* L. Methanol Extract. *Pharmaceuticals* **17**, 121 (2024). 38256954 10.3390/ph17010121 PMC10819509

[57] Pagnotta, E. et al. Glucosinolates in Reseda lutea L.: Distribution in plant tissues during flowering time. *Biochem. Syst. Ecol.* **90**, 104043. (2020). 10.1016/j.bse.2020.104043

[58] Rather, M. A., Dar, B. A., Sofi, S. N., Bhat, B. A. & Qurishi, M. A. Foeniculum vulgare: A comprehensive review of its traditional use, phytochemistry, pharmacology, and safety. *Arab. J. Chem.* **9**, 1574–1583. (2012). 10.1016/j.arabjc.2012.04.011

[59] Dugo, G. et al. Characterization of cold-pressed and processed bergamot oils by using GC-FID, enantio-GC, MDGC, HPLC and HPLC-MS-IT-TOF.* J. Essential Oil Res.*** 24**(2):93–117. (2012). 10.1080/10412905.2012.659526

[60] Zidorn, C. et al. Polyacetylenes from the Apiaceae vegetables carrot, celery, fennel, parsley, and parsnip and their cytotoxic activities. *J. Agric. Food Chem.* **53** (7), 2518–2523. (2005). 10.1021/jf048041s 15796588 10.1021/jf048041s

[61] Salatino, A., Salatino, M. L. F. & Negri, G. Traditional uses, chemistry and pharmacology of Croton species (Euphorbiaceae). *J. Braz. Chem. Soc.* **18** (1), 11–33. (2007). 10.1590/s0103-50532007000100002

